# Honokiol and Magnolol: Insights into Their Antidermatophytic Effects

**DOI:** 10.3390/plants10112522

**Published:** 2021-11-19

**Authors:** Adriana Trifan, Andra-Cristina Bostănaru, Simon Vlad Luca, Veronika Temml, Muhammad Akram, Sonja Herdlinger, Łukasz Kulinowski, Krystyna Skalicka-Woźniak, Sebastian Granica, Monika E. Czerwińska, Aleksandra Kruk, Hélène Greige-Gerges, Mihai Mareș, Daniela Schuster

**Affiliations:** 1 Department of Pharmacognosy, Faculty of Pharmacy, “Grigore T. Popa” University of Medicine and Pharmacy Iasi, 700115 Iasi, Romania; adriana.trifan@umfiasi.ro; 2 Laboratory of Antimicrobial Chemotherapy, Faculty of Veterinary Medicine, “Ion Ionescu de la Brad” Iasi University of Life Sciences, 700489 Iasi, Romania; acbostanaru@gmail.com; 3 Biothermodynamics, TUM School of Life and Food Sciences, Technical University of Munich, 85354 Freising, Germany; 4 Department of Pharmaceutical Chemistry, Paracelsus Medical University Salzburg, 5020 Salzburg, Austria; veronika.temml@pmu.ac.at (V.T.); muhammad.akram@pmu.ac.at (M.A.); sonja.herdlinger@pmu.ac.at (S.H.); daniela.schuster@pmu.ac.at (D.S.); 5 Department of Natural Products Chemistry, Medical University of Lublin, 20-093 Lublin, Poland; lukaszkulinowski@gmail.com (Ł.K.); kskalicka@pharmacognosy.org (K.S.-W.); 6 Microbiota Lab, Centre for Preclinical Studies, Department of Pharmacognosy and Molecular Basis of Phytotherapy, Medical University of Warsaw, 02-097 Warsaw, Poland; sgranica@wum.edu.pl (S.G.); akruk@wum.edu.pl (A.K.); 7 Department of Biochemistry and Pharmacogenomics, Faculty of Pharmacy, Medical University of Warsaw, 02-097 Warsaw, Poland; monika.czerwinska@wum.edu.pl; 8 Centre for Preclinical Research, Medical University of Warsaw, 02-097 Warsaw, Poland; 9 Bioactive Molecules Research Laboratory, Department of Chemistry and Biochemistry, Faculty of Sciences, Section II, Lebanese University, Jdeidet el-Matn B.P. 90656, Lebanon; helenegreige73@gmail.com

**Keywords:** ergosterol, checkerboard assay, cytokines, squalene, synergy, terbinafine, *Trichophyton rubrum*

## Abstract

Dermatophyte infections represent a significant public health concern, with an alarming negative impact caused by unsuccessful therapeutic regimens. Natural products have been highlighted as a promising alternative, due to their long-standing traditional use and increasing scientific recognition. In this study, honokiol and magnolol, the main bioactives from *Magnolia* spp. bark, were investigated for their antidermatophytic activity. The antifungal screening was performed using dermatophyte standard strains and clinical isolates. The minimal inhibitory concentration (MIC) and the minimal fungicidal concentration (MFC) were determined in accordance with EUCAST-AFST guidelines, with minor modifications. The effects on ergosterol biosynthesis were assessed in *Trichophyton rubrum* cells by HPLC-DAD. Putative interactions with terbinafine against *T. rubrum* were evaluated by the checkerboard method. Their impact on cells’ viability and pro-inflammatory cytokines (IL-1β, IL-8 and TNF-α) was shown using an ex vivo human neutrophils model. Honokiol and magnolol were highly active against tested dermatophytes, with MIC and MFC values of 8 and 16 mg/L, respectively. The mechanism of action involved the inhibition of ergosterol biosynthesis, with accumulation of squalene in *T. rubrum* cells. Synergy was assessed for binary mixtures of magnolol with terbinafine (FICI = 0.50), while honokiol-terbinafine combinations displayed only additive effects (FICI = 0.56). In addition, magnolol displayed inhibitory effects towards IL-1β, IL-8 and TNF-α released from lipopolysaccharide (LPS)-stimulated human neutrophils, while honokiol only decreased IL-1β secretion, compared to the untreated control. Overall, honokiol and magnolol acted as fungicidal agents against dermatophytes, with impairment of ergosterol biosynthesis.

## 1. Introduction

Dermatophytosis has emerged as an important public health issue, affecting up to 25% of the global population [1]. Dermatophytes are filamentous fungi that invade the keratinized structures of the skin, hair and nails, causing infections with different degrees of inflammation among immunocompetent individuals [2]. According to de Hoog et al. [3], dermatophytes are classified into seven clades, with prevalence in clinical isolates of *Trichophyton, Epidermophyton, Microsporum* and *Nannizzia* genera. *Trichophyton* species represent the major causative agents of dermatophytosis, with *Trichophyton rubrum* being responsible for up to 70% of infections of the feet, nails and body [4]. Although several classes of antifungal drugs are available (e.g., allylamines, azoles and morpholine derivatives), their clinical use is hampered by side effects, long-term treatment regimens and emergence of fungal resistance [5,6]. Therefore, the search for novel antifungal agents targeting structures that are unique to dermatophytes is highly imperative.

Natural products were highlighted as promising alternatives or complementary agents in dermatophytosis, due to their long-standing traditional use and increasing scientific recognition. Among the plant-derived products, phenolic compounds are a class of specialized metabolites that possess significant antifungal activity and, in particular, their antidermatophytic effects have been proven through numerous in vitro and in vivo studies [7,8]. 

**Figure 1:**
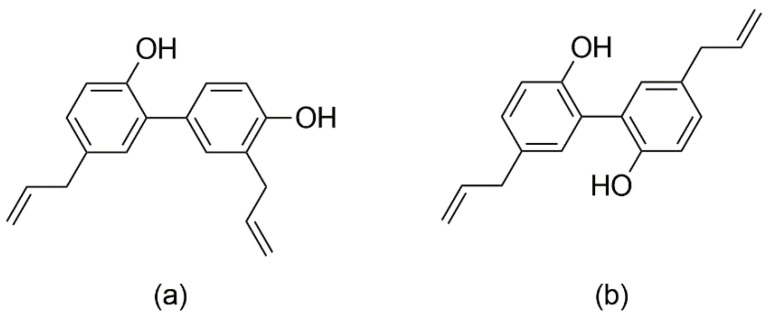
Chemical structures of honokiol (**a**) and magnolol (**b**).

Honokiol and magnolol are the main phenolics found in the bark of *Magnolia officinalis* Rehder & E.H. Wilson, species used as a remedy in the Chinese and Japanese Traditional Medicines to alleviate gastrointestinal disorders, anxiety, cough and allergic rhinitis [9]. Honokiol (5,3′-diallyl-2,4′-dihydroxybiphenyl) and magnolol (5,5′-diallyl-2,2′-dihydroxybiphenyl) are neolignan isomers, composed of two phenylpropanoid units linked by an aromatic C-C bond (Figure 1). The literature abounds in reports assessing their properties, e.g., anticancer, neuroprotective, cardioprotective, antimicrobial and anti-inflammatory effects [10,11,12,13]. Both compounds have good safety profiles, with no reported mutagenic or genotoxic effects [14]. Honokiol and magnolol have been promoted as promising antifungal agents against various human and plant pathogens [15,16,17,18]. Still, the data regarding the susceptibility of dermatophytes to honokiol and magnolol are scarce, with only several studies reporting the in vitro efficacy towards *Trichophyton mentagrophytes* and *Microsporum gypseum* clinical isolates [19,20]. Moreover, there are no literature reports on their effects against *T. rubrum*, the main causative agent of dermatophytosis.

Our study aims to assess the antidermatophytic potential of honokiol and magnolol, with a special focus on *T. rubrum*. In this respect, honokiol and magnolol were investigated for their antifungal effects on dermatophytes, both standard strains and clinical isolates. Further, the interference of honokiol and magnolol in the ergosterol pathway and putative interactions with terbinafine were evaluated using *T. rubrum* as a model microorganism. In addition, their impact on the pro-inflammatory secretion of cytokines in an ex vivo human neutrophils model was assessed. 

## 2. Results

### 2.1. Antifungal Susceptibility Results

The antifungal activity of honokiol and magnolol was assessed using the microdilution method. The minimal inhibitory concentration (MIC) and minimal fungicidal concentration (MFC) values obtained against dermatophyte strains are shown in Table 1. Honokiol and magnolol displayed similar activity against investigated dermatophyte strains, inhibiting mycelial growth at MIC values of 8 mg/L (30.03 µM). Compared to the investigated neolignans, terbinafine was highly active against dermatophyte strains (MIC range of 0.031–0.062 mg/L). Furthermore, the MFC values revealed that both honokiol and magnolol exhibited fungicidal activity against the tested dermatophytes (Table 1).

### 2.2. Effects on Ergosterol Biosynthesis

**Figure 2:**
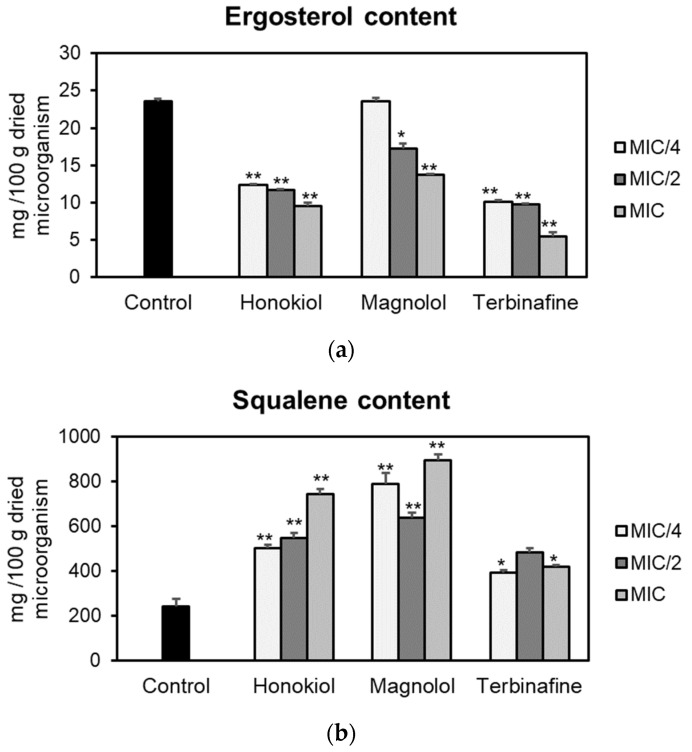
Ergosterol (**a**) and squalene (**b**) content (mg/100 g dried microorganism) in *T. rubrum* ATCC 28188 cells after treatment with honokiol, magnolol and terbinafine (positive control) at MIC/4, MIC/2 and MIC values. Data were expressed as mean ± SD of three separate experiments. Statistical significance: * *p* < 0.01, ** *p* < 0.001 compared to the untreated control.

The effects of honokiol and magnolol on the ergosterol pathway were assessed using *T. rubrum* ATCC 28188 as a model microorganism. Fungal cells were cultured with different concentrations of the test compounds (MIC/4, MIC/2, MIC), followed by sterols’ extraction and quantification of ergosterol and squalene by an HPLC-DAD method. The obtained results are presented in Figure 2. Honokiol and magnolol significantly reduced the ergosterol amount in *T. rubrum* cells, following a concentration-dependent pattern. Moreover, at MIC values, honokiol had a higher degree of potency in inhibiting ergosterol biosynthesis compared to magnolol (59.59% vs. 41.68% decrease compared to the untreated control). Still, positive control terbinafine impaired ergosterol production to a higher extent than investigated neolignans, with a 76.52% decrease relative to the untreated control (Figure 2a).

Squalene, the first intermediate in the ergosterol pathway, was further quantified. The effects of honokiol and magnolol upon squalene production are depicted in Figure 2b. Squalene levels were significantly altered when *T. rubrum* was exposed to different concentrations of neolignans. At MIC values, honokiol and magnolol treatment markedly increased squalene synthesis in *T. rubrum* cells, by 205.19% and 266.35% respectively, relative to the untreated control. This pattern was similar to the positive control terbinafine, that also significantly induced squalene accumulation in fungal cells (Figure 2b). 

### 2.3. Molecular Docking Results

**Figure 3:**
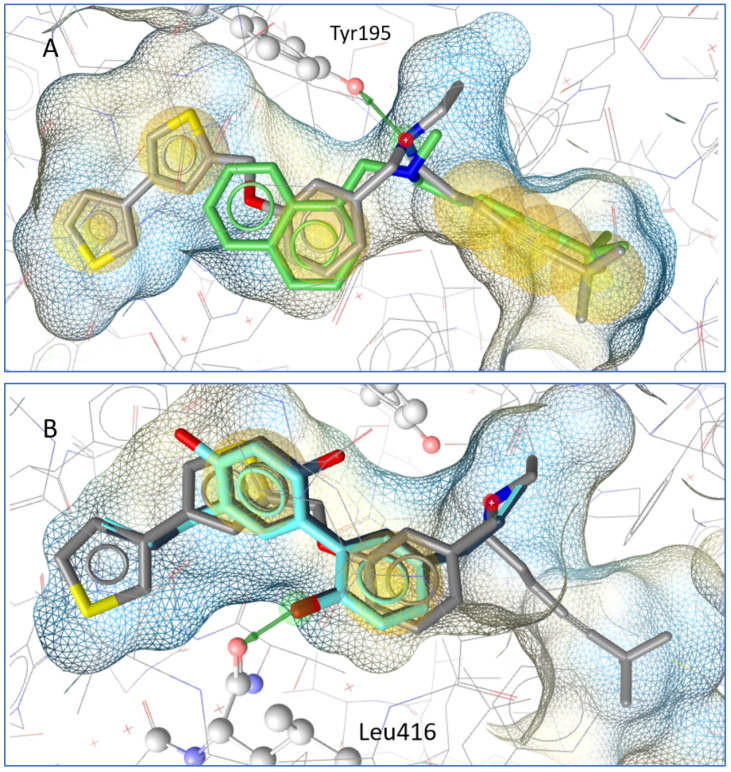
(**A**) Docking pose of terbinafine (green) in comparison to the co-crystallized molecule NB-598. A hydrogen bond (green arrow) is formed to Tyr195 from the squalene epoxidase. (**B**) Magnolol (dark blue) and honokiol (light blue) in comparison with NB-598. Both form a hydrogen bond with Leu 416 from the squalene epoxidase.

In a molecular docking simulation, honokiol and magnolol were compared to terbinafine and the co-crystallized squalene epoxidase inhibitor NB-598. A key interaction in the binding pocket is a hydrogen bridge to Tyr195 from the squalene epoxidase that is formed by inhibitors such as NB-598 and terbinafine. Honokiol and magnolol do not form the same interactions, but their aromatic systems are in a similar location to the co-crystallized inhibitor; in addition, both neolignans form a hydrogen bond with Leu416 from the squalene epoxidase (see Figure 3).

### 2.4. Checkerboard Assay Results

The evaluation of in vitro interactions between honokiol or magnolol and terbinafine was subsequently assessed by the checkerboard microtiter assay. Considering its high sensitivity to terbinafine, in vitro experiments using *T. rubrum* ATCC 28188 were undertaken. The combinatorial effects of the investigated compounds with terbinafine are reported in Table 2. 

The checkerboard testing revealed that the binary associations of magnolol with terbinafine were synergistic against *T. rubrum*, with a fractional inhibitory concentration index (FICI) value of 0.50. Magnolol significantly potentiated the activity of terbinafine in combinatorial therapy, with a 4-fold reduction of its MIC, from 0.031 to 0.007 mg/L. On the other hand, honokiol showed only additive effects in combination with terbinafine (FICI value of 0.56). Moreover, the MIC of both neolignans decreased in combination with terbinafine: a 16-fold reduction in the case of honokiol, while the MIC of magnolol was only subject to a 4-fold reduction. 

### 2.5. Effects on Cytokine Production in Human Neutrophils

Based on the MIC values, honokiol and magnolol (concentration range 12.5–50 µM) were further assessed for their ability to influence the viability of human neutrophils and the production of pro-inflammatory cytokines in lipopolysaccharide (LPS)-stimulated neutrophils.

#### 2.5.1. Effects on Neutrophils’ Viability

**Figure 4:**
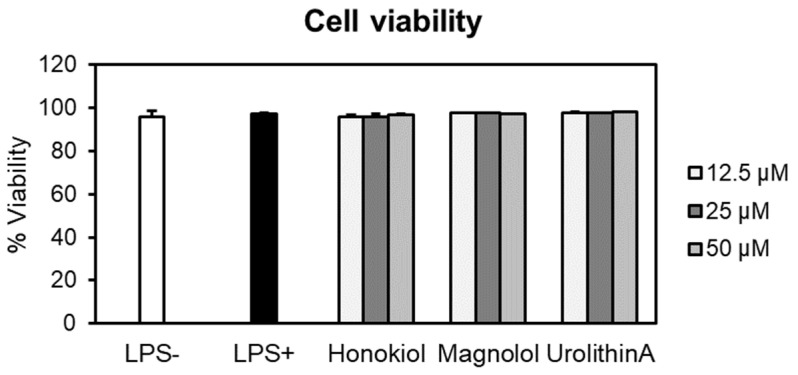
Neutrophils’ viability (%) after 24 h treatment with honokiol, magnolol and urolithin A (12.5, 25 and 50 μM). Results were expressed as mean ± SEM of three separate experiments performed with cells isolated from four independent donors. LPS—lipopolysaccharide.

At the tested concentrations, neither honokiol nor magnolol induced cytotoxic effects in human neutrophils compared to negative control cells (LPS–) (Figure 4). For example, at the highest concentration, the cell viabilities were 96.65% for honokiol and 97.22% in the case of magnolol. It is worth noting that the viability of the cells treated with test compounds was comparable to that of LPS-stimulated cells (97.18%). Furthermore, the treatment with urolithin A, used as the positive control in the following cytokine production assays, did not display cytotoxic effects towards neutrophils (viability of neutrophils 97.63–98.06%). Meanwhile, Triton X-100 (0.1%) caused a very strong reduction of the cell viability, with only 1.51% of viable cells (data not shown).

#### 2.5.2. Effects on IL-1β, IL-8 and TNF-α Secretion

**Figure 5:**
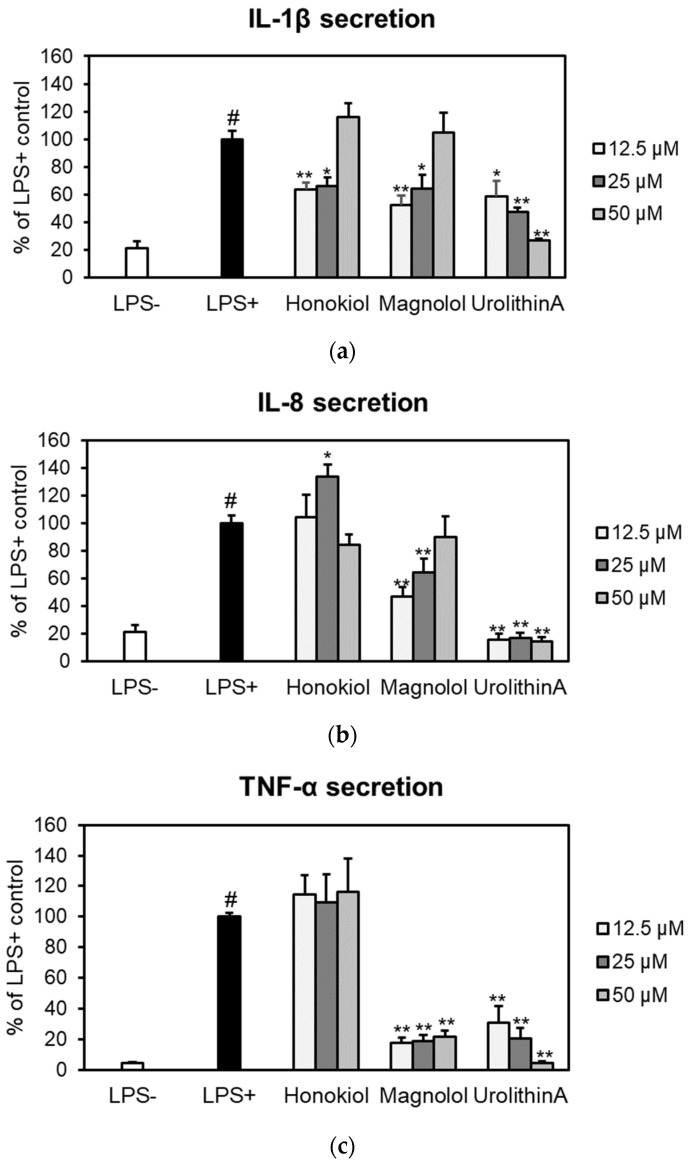
Effects of honokiol, magnolol and urolithin A (12.5, 25 and 50 μM) on IL-1β (**a**), IL-8 (**b**) and TNF-α (**c**) release in LPS-stimulated neutrophils. Results were expressed as mean ± SEM of three separate experiments performed with cells isolated from four independent donors. Statistical significance: # *p* < 0.001 compared to the non-stimulated control (LPS–), and * *p* < 0.01, ** *p* < 0.001 compared to the stimulated control (LPS+).

The effects of honokiol and magnolol on the secretion of pro-inflammatory cytokines (IL-1β, IL-8 and TNF-α) were assessed by ELISA in an ex vivo human blood neutrophils model (Figure 5). 

Both compounds significantly reduced IL-1β release from LPS-stimulated neutrophils, with honokiol displaying a slightly higher inhibition of IL-1β production at concentrations of 12.5 and 25 µM (Figure 5a). It is worth mentioning that, at 12.5 µM, the potency of magnolol (52.56%) was similar to that of the positive control urolithin A, which decreased the IL-1β secretion to 58.57% of the LPS+ control. The effects of honokiol or magnolol on IL-8 and TNF-α secretion are depicted in Figure 5b,c. Only magnolol was found active within the tested concentration range. At 12.5 μM, it was able to decrease IL-8 levels up to 47.14% when compared to LPS-stimulated neutrophils. Moreover, magnolol significantly displayed TNF-α inhibitory effects (17.59% of LPS+ control, at 12.5 μM), while similar effects were shown for the positive control urolithin A at 25 μM (20.14% of LPS+ control).

## 3. Discussion

The Plant Kingdom represents a vast reservoir of metabolites endowed with antifungal properties, including antidermatophytic effects, as proven in the in vitro, in vivo and clinical settings of dermatophytosis [7,8,21,22]. Our study was designed to assess the activity of honokiol and magnolol against dermatophytes, with insight into their mechanism of action and putative interactions with terbinafine, using *T. rubrum* as a model microorganism. 

The antifungal effect of honokiol and magnolol was tested using the broth microdilution method against standard strains and clinical isolates of dermatophytes (standard strains, *T. rubrum* ATCC 28188 and *T. mentagrophytes* ATCC 9533, and clinical isolates of *T. rubrum, T. ajelloi, M. gypseum* and *M. canis*). According to Kuete et al. [23], the antimicrobial potential of natural compounds is correlated with their MIC values, as follows: significant activity (MIC < 10 mg/L), moderate (10 < MIC ≤ 100 mg/L) and low or negligible (MIC > 100 mg/L). Thus, honokiol and magnolol were highly active against the tested fungi (MIC 8 mg/L), justifying their putative use as stand-alone antimicrobial agents. The data regarding the antidermatophytic activity of honokiol and magnolol are scarce, with only two studies having assessed such properties. Thus, Bang et al. [20] found that both compounds inhibited the growth of clinical isolates of *T. mentagrophytes* and *M. gypseum*, with MIC values of 25 mg/L for honokiol and 50 mg/L in the case of magnolol. Kim et al. [19] showed that honokiol inhibited the fungal growth of *T. mentagrophytes* KCTC6077 (MIC = MFC = 13.32 mg/L). Still, a direct comparison between the MIC values obtained in the present study and the literature data is hampered by the implementation of different protocols (e.g., obtaining the serial dilutions, fungi growth phase and vitality, inoculum preparation and volume, culture medium and pH, temperature and incubation time). To the best of our knowledge, this is the first report on the antifungal effects of honokiol and magnolol against *T. rubrum*, *T. ajelloi* and *M. canis.* In addition, since MFC and MIC values were identical for both honokiol and magnolol, it can be concluded that they exhibit a fungicidal activity against the tested dermatophytes. This could be of utmost importance in the clinical settings of recurrent and multi-drug-resistant dermatophytoses, as the use of fungistatic agents is commonly associated with fungal resistance [8,24].

In order to gain insight into the mechanism of antifungal activity, the influence of honokiol and magnolol on ergosterol biosynthesis in *T. rubrum* cells was evaluated. Thus, the amount of ergosterol was quantified in fungal cells exposed to increasing concentrations of honokiol and magnolol (MIC/4, MIC/2, MIC). It was observed that both neolignans significantly decreased the ergosterol content compared to the untreated control, following a concentration-dependent pattern. Nevertheless, honokiol displayed a higher degree of potency relative to magnolol in inhibiting ergosterol production. In addition, the treatment of *T. rubrum* cells with the positive control terbinafine showed a similar trend, with a concentration-dependent decrease of the ergosterol content (Figure 2a). Moreover, squalene, the first intermediate in the ergosterol biosynthetic pathway, was also quantified. Our investigation showed that, following exposure to either honokiol, magnolol or terbinafine, there was a significant increase in squalene production in *T. rubrum* cells (Figure 2b). In addition, squalene was detected in higher amounts in order of size when compared to ergosterol (Figure 2). It is known that terbinafine inhibits ergosterol synthesis by targeting squalene epoxidase, a key enzyme that catalyzes squalene conversion to 2,3-oxidosqualene [25]. Consequently, ergosterol deficiency interferes with the membrane’s function and cell growth (fungistatic effect), while squalene accumulation entails deposition of lipid vesicles that lead to the disruption of the fungal membrane (fungicidal effect) [26,27]. Our results confirm that terbinafine inhibits ergosterol synthesis, with an accumulation of squalene in *T. rubrum* cells. Since honokiol and magnolol showed a similar pattern to terbinafine, it can be hypothesized that both compounds might interfere in the ergosterol pathway at the same limiting step, namely squalene conversion into 2,3-oxidosqualene, with subsequent accumulation of the first in fungal cells. Molecular docking studies were further undertaken in order to investigate their potential binding to *T. rubrum* squalene epoxidase. Our experiment showed that honokiol and magnolol fit the binding site of the enzyme in the same location as the co-crystallized inhibitor NB-598 (Figure 3B). Both neolignans displayed similar interactions with the binding pocket via hydrogen bonding to Leu416 catalytic residue, while terbinafine formed a hydrogen bridge to Tyr195 (Figure 3A,B). This might explain the different degrees of potency exhibited by neolignans relative to terbinafine in impacting the ergosterol synthesis. Thus, the in silico study supports the hypothesis of inhibition of *T. rubrum* squalene epoxidase by honokiol and magnolol. 

Furthermore, the interactions between terbinafine and the investigated neolignans were assessed by the checkerboard method, using *T. rubrum* as a model microorganism. Our investigation showed synergistic interactions between magnolol and terbinafine (FICI = 0.50), while honokiol only displayed additive effects when combined with terbinafine against *T. rubrum* (FICI = 0.56). It is noteworthy that, at lower sub-inhibitory concentrations (MIC/4), magnolol induced a 4-fold enhancement of terbinafine’s activity against *T. rubrum* (Table 2). The observed outcome may be due to the ability of honokiol and magnolol to interfere with the ergosterol pathway, causing the disruption and subsequent permeability loss of the fungal membrane. Moreover, these changes could facilitate the terbinafine entry into the cells with a pronounced impairment of ergosterol biosynthesis. Still, additional experiments are needed in order to fully elucidate the mechanism underlying the synergistic and additive effects of such combinations. Indeed, honokiol and magnolol displayed similar fungicidal potency and interfered in the ergosterol pathway of *T. rubrum*, but the differences assessed by the checkerboard method could reside in their structural features. Even though honokiol and magnolol are isomers (Figure 1), the position of aromatic hydroxyls and allyl groups could influence their ability to modulate different targets of *T. rubrum* metabolism and pathogenicity. 

Combination therapy associating antifungal drugs is already used to improve the monotherapy results in clinical settings of refractory dermatophytosis [28,29]. In addition, combinatorial strategies associating conventional drugs (e.g., terbinafine) and plant phenolics have already been proposed as a complementary therapy against dermatophytes [21,30]. Numerous in vitro studies have demonstrated the antidermatophytic properties of phenolic compounds, as their mechanism relies on the disruption of the cell wall and membrane, the inhibition of spore germination, oxidative stress induction, the inhibition of detoxifying enzymes, mitochondrial respiration, ergosterol and cellular proteins’ synthesis, efflux pumps and biofilm formation [2,31]. Even though terbinafine is the preferred treatment in *T. rubrum*-related dermatophytosis, its use is hampered by side effects, hepatoxicity, drug interactions, patient co-morbidities and fungal resistance [5,32,33]. In this context, magnolol-terbinafine synergistic combinations might increase the potency of the antifungal drug with a reduction of effective doses, consequently minimizing its side effects and toxicity. Compared to other molecules containing aromatic rings, the flexibility of aromatic linkage of biphenyls such as magnolol (Figure 1) enables various interactions with the proteins’ surface [17]. Thus, the binary mixture of magnolol-terbinafine could display a multi-targeted activity, decreasing the risk of the emergence of fungal resistance. 

Herein, we reported for the first time the combinatorial effects of honokiol and magnolol with terbinafine against *T. rubrum*. Previously, both honokiol and magnolol were shown to synergize with azoles (e.g., fluconazole) in in vitro models of candidiasis. The mechanism of activity consisted in targeting the virulence factors and resistance mechanisms of *Candida* spp., such as cell adhesion, transition from yeast to hyphae, biofilm formation and the ergosterol pathway [34,35].

Based on the MIC values, the influence of honokiol and magnolol on the pro-inflammatory cytokines’ release in ex vivo LPS-stimulated human neutrophils was evaluated. Neutrophils are the first line of host defense against *T. rubrum*, as clinical setups revealed a dense infiltration of neutrophils in infected areas [36]. After recruitment from the bloodstream, the activation of neutrophils in response to fungi attack includes phagocytosis, proteases secretion, reactive oxygen species production, alongside the release of extracellular traps, pro-inflammatory cytokines (e.g., TNF-α, IL-1β, IL-6 and IL-8), chemokines and growth factors [37,38]. Still, the prolonged activation of neutrophils hinders the resolution of fungal infection, sustaining a chronic inflammation that can, in turn, contribute to the colonization of the neighboring tissue [39]. Thus, therapeutic agents endowed with dual activity, namely selective antifungal and anti-inflammatory effects, are preferred to modulate the balance between pro- and anti-inflammatory signals in human host–dermatophyte interactions. Moreover, the anti-inflammatory properties might support lesion healing and alleviate symptoms related to dermatophytosis [40].

The putative cytotoxic effects of honokiol and magnolol (concentration range of 12.5–50 µM) were evaluated towards human neutrophils obtained ex vivo from healthy volunteers. Neither neolignans altered neutrophils viability, as no toxicity was recorded at the tested concentrations (Figure 4), underlying their safety in terms of pharmaceutical use. In addition, the neutrophils displayed good viability and the LPS-stimulation markedly increased the release of the pro-inflammatory cytokines IL-1β, IL-8 and TNF-α (Figure 5). Our data revealed that the treatment with honokiol and magnolol (24 h incubation) inhibited the cytokines’ generation in LPS-stimulated neutrophils to different degrees. Both compounds reduced IL-1β production, with honokiol displaying a slightly stronger inhibition when compared to magnolol (Figure 5a). Regarding IL-8 and TNF-α, only magnolol significantly lowered their secretion in stimulated human neutrophils (Figure 5b,c). The ability of honokiol and magnolol to inhibit the release of pro-inflammatory cytokines is well-known, and derives from in vitro, ex vivo and in vivo studies [11,12]. Nonetheless, by comparing our results with those obtained in similar experimental models, one must note that the outcomes vary depending on several factors, such as the cell type, the agent used for cytokine generation and the treatment conditions (honokiol and magnolol concentrations, and period of incubation). For example, Wang et al. [41] assessed the potency of honokiol to inhibit IL-1β and IL-8 generation by TNF-α in an ex vivo human neutrophils model. After a 24 h incubation with honokiol (up to 10 µM), a concentration-dependent inhibition of IL-1β and IL-8 expression was observed. Additionally, Li et al. [42] demonstrated that honokiol was able to inhibit IL-1β and TNF-α release in ex vivo LPS-activated human monocyte-derived dendritic cells, and treatment for 48 h with honokiol (concentration range of 18.75–75 µM) decreased both cytokines’ production compared to the untreated control. Liu et al. [43] showed that magnolol at 10 µM reduced IL-8 generation in ex vivo human gingival fibroblasts upon stimulation with advanced glycation end products.

Overall, considering the observed antidermatophytic activity, alongside the inhibitory effects of pro-inflammatory cytokine release, the further development of formulations including honokiol, magnolol and their combinations with terbinafine is justified. Still, like other natural phenolics, the pharmaceutical application of honokiol and magnolol could be hampered by their low water solubility and chemical instability [14]. Hence, the inclusion in delivery systems such as cyclodextrins, micelles and liposomes could be an approach towards overcoming such limitations [11,44,45]. Besides, the mode of administration impacts the availability of honokiol and magnolol at the infection site. Thus, a topical use of both neolignans and their combinations with terbinafine is easily envisioned. Notwithstanding our observations, additional studies are needed to assess whether the in vitro results translate into similar outcomes in in vivo settings and to fully elucidate the mechanism of antidermatophytic activity. 

## 4. Materials and Methods

### 4.1. Chemicals

4-(2-hydroxyethyl)-1-piperazineethanesulfonic acid) (HEPES), 3-(N-morpholino) propanesulphonic acid (MOPS), acetonitrile, anhydrous glucose, chloramphenicol, cycloheximide, dimethyl sulfoxide (DMSO), dextran, ergosterol, ethanol, ethyl acetate, fetal bovine serum (FBS), formic acid, hexane, honokiol, L-glutamine, magnolol, methanol, potassium hydroxide, propidium iodide (PI), RPMI 1640 medium, squalene, and terbinafine hydrochloride were acquired from Sigma-Aldrich (Steinheim, Germany). Phosphate buffered saline (PBS) was purchased from Gibco (Gibco, HK, China) and (Ca^2+^)-free PBS from Biomed (Lublin, Poland). Pancoll Human (1.077 g/mL) was bought from GmbHPAN-Biotech (Aidenbach, Germany). Penicillin and streptomycin were acquired from Biowest (Nauillé, France). Lipopolysaccharide (LPS) from *Escherichia coli* 0111:B4 was purchased from Merck (Kenilworth, NJ, USA). Human ELISA sets (IL-1β, IL-8, TNF-α) were bought from BD Biosciences (Franklin Lake, NJ, USA). Sabouraud dextrose agar (SDA) and potato dextrose agar (PDA) were from Biolab (Budapest, Hungary).

### 4.2. Plant Material, Extraction

*Magnolia officinalis* barks were purchased from a local supplier (Chinasan BV, Heerlen, The Netherlands) and identified by one of the authors (A.T.). A voucher specimen (MO1004/2020) has been stored in the Department of Pharmacognosy, “Grigore T. Popa” University of Medicine and Pharmacy Iasi (Romania). The powdered bark (100 g) was extracted with ethanol (3 × 1000 mL) in an ultrasound bath for 30 min. After solvent removal, 5.89 g of crude extract was obtained.

### 4.3. Isolation of Honokiol and Magnolol

Honokiol (18 mg, >95% HPLC-DAD purity) and magnolol (30 mg, >95% HPLC-DAD purity) were isolated from the crude extract with a centrifugal partition chromatography unit (CPC250, Gilson, Middleton, WI, USA). The following separation conditions were used: hexane/ethyl acetate/methanol/water (7/3/7/3, *v*/*v*/*v*/*v*), descending mode (upper phase as mobile phase), rotation speed 2700 rpm, flow rate 6 mL/min, injection volume 10 mL, sample concentration 30 mg/mL (dissolved in lower phase), CPC column volume 250 mL, stationary phase retention 68% and UV wavelength 254 nm. 

### 4.4. Microbial Strains

Two type strains (*Trichophyton rubrum* ATCC 28188 and *Trichophyton mentagrophytes* ATCC 9533) and five clinical isolates of dermatophytes (*Microsporum gypseum* RTCC 2172, *Microsporum canis* RTCC 1883, *Trichophyton ajelloi* RTCC 1356, *Trichophyton rubrum* RTCC 1348 and *Trichophyton rubrum* RTCC 2158) isolated from human nail and skin infections were used as testing microorganisms (ATCC American Type Culture Collection; RTCC: Romanian Type Culture Collection). The strains were stored at −80 °C in Sabouraud dextrose broth with glycerol. Prior to testing, the strains were sub-cultured in PDA with chloramphenicol to ensure optimal growth, followed by an incubation of up to 15 days at 28 °C. All strains were checked during the incubation period, and they were used when a maximal number of conidia were formed.

### 4.5. Antifungal Susceptibility Testing

The in vitro antifungal susceptibility of dermatophyte strains was assessed by the broth microdilution method using 96-well plates, in accordance with The European Committee on Antimicrobial Susceptibility Testing Subcommittee on Antifungal Susceptibility Testing (EUCAST-AFST) guidelines [46], with slight modifications following the methodology described by Saunte et al. [47] and Markantonatou et al. [48]. Serial double dilutions of honokiol and magnolol ranging from 1 to 512 mg/L were prepared into RPMI 1640 medium 2% glucose buffered with 0.165 M MOPS and supplemented with chloramphenicol 50 mg/L and cycloheximide 300 mg/L, followed by inoculation (10^5^ CFU/mL). The final concentration of DMSO did not exceed 1%. Terbinafine was used as a reference drug (concentration range 0.015–8 mg/L). Fungal growth control and sterility control were used in order to ensure the reliability of the tests. The incubation was performed at 28 °C for 5 days. The MIC was considered as the lowest concentration with no visual growth of the tested fungi. The MFC was assessed by inoculating 10 µL from wells that did not show growth in the MIC assay onto SDA plates, followed by incubation at 28 °C for 72 h. The MFC was considered as the lowest concentration with no growth or less than five colonies in the case of subculture (corresponding to ~99.9% killing activity). All experiments were run in triplicate.

### 4.6. Evaluation of Effects on Ergosterol Biosynthesis

#### 4.6.1. Sterols’ Extraction

The dilutions of honokiol and magnolol prepared into RPMI medium as mentioned above (25 mL, concentrations of MIC/4, MIC/2, MIC) were inoculated with cell suspensions of *T. rubrum* ATCC 28188 (25 mL, 10^5^ CFU/mL). A positive control (terbinafine) and negative control (an untreated sample) were used. Cultures were incubated with shaking at 28 °C for 5 days. After incubation, sterols were extracted by saponification, following a previously described method [49,50], with slight modifications. In summary, fungal cells were separated by centrifugation (3000 rpm, 5 min), washed twice with sterile distilled water, dried over night at 40 °C and weighted. Cell pellets were added with 3 mL of 25% alcoholic potassium hydroxide, transferred to sterile borosilicate glass screw-cap tubes and vigorously vortexed, followed by water bath incubation (85 °C, 60 min). After cooling, sterols were extracted twice with *n*-hexane (2 mL) by vigorous vortex mixing. The organic phase was evaporated to dryness and subsequently redissolved in methanol for the HPLC-DAD analysis.

#### 4.6.2. HPLC-DAD Quantification of Ergosterol and Squalene 

The quantification of ergosterol and squalene was performed with a Shimadzu HPLC (Shimadzu, Tokyo, Japan) equipped with a binary pump (LC-20AB), degasser (DGU-20A3), auto-sampler (SIL-20A) and a diode array detector (SPD-M20A). The separations were carried out according to the method of Lopes et al. [50], with slight modifications. Briefly, a Dionex Acclaim 120 C18 (4.6 × 250 mm, 5 μm) column with a mobile phase consisting of methanol/acetonitrile (35/65, *v*/*v*) was used. The run-time, flow rate and injection volume were 45 min, 0.8 mL/min and 10 μL, respectively. Squalene was analyzed at 205 nm, while ergosterol at 280 nm. Adequate calibration curves for squalene (linearity range 2–20 μg/mL; regression equation: y = 21903.1x − 5877.7; correlation coefficient (*r*) *=* 0.9999; limit of detection (LOD) = 0.11; limit of quantification (LOQ) = 0.33) and ergosterol (linearity range 2–20 μg/mL; regression equation: y = 21229.8x − 3860.5; *r =* 0.9999; LOD = 0.05; LOQ = 0.15) were performed. LOD was calculated as 3.3 × σ/S, whereas LOQ as 10 × σ/S, with σ representing the standard deviation of the y-intercept and S the slope. Triplicate injections were carried out for each standard and sample. The results were expressed as mg/100 g dried weight of the microorganism.

### 4.7. Molecular Docking Studies

Molecular docking simulations were conducted in Gold 5.2. (C.C.D.C., Cambridge, UK). The Pdb structure 6C6P of squalene epoxidase co-crystallized with NB-598 was selected as a target structure [51]. In preparation, all water was deleted from the structure and hydrogen atoms were added. The binding site was defined in an 8 Å radius around the co-crystalized ligand and GoldScore (https://www.ccdc.cam.ac.uk, accessed on 24 October 2021) was selected as a scoring function. The protein–ligand interactions of the docking poses were analyzed using LigandScout 4.2 (www.inteligand.com/ligandscout, accessed on 25 October 2021). In order to validate the docking settings, a redocking with NB-598 was conducted. Under the employed settings, an RMSD of 0.457 to the co-crystallized conformation was found.

### 4.8. Checkerboard Assay

The checkerboard broth microdilution method was used to evaluate the interactions between honokiol or magnolol and terbinafine against *T. rubrum* ATCC 28188. Serial double dilutions of compounds and terbinafine were prepared as previously described to determine the MIC values. Honokiol or magnolol were dispensed in increasing concentrations along the X-axis (MIC/128 − 4 × MIC), while terbinafine was dispensed in increasing concentrations along the Y-axis (MIC/16 − 4 × MIC), followed by inoculation (10^5^ CFU/mL) [47,52]. The plates were incubated at 28 °C for 5 days. Assays were performed in duplicate and repeated thrice. The fractional inhibitory concentration index (FICI) was calculated using the following equation:

FICI = FIC _compound_ + FIC _terbinafine_,

where FIC _compound_ = MIC _compound in combination_/MIC _compound alone_ and FIC _terbinafine_ = MIC _terbinafine in combination_ / MIC _terbinafine alone_. 

The interactions between the compounds and terbinafine were interpreted based on the FICI values. Synergy was defined by FICI ≤ 0.5, addition was considered when 0.5 > FICI ≤ 1, indifference if 1 > FICI ≤ 4 and antagonism for FICI > 4 [53,54].

### 4.9. Evaluation of Cytokine Production in Human Neutrophils

#### 4.9.1. Neutrophils’ Isolation

The protocol used to prepare the buffy coat fractions complied with the principles stated in the Declaration of Helsinki. Peripheral venous blood for buffy coats was collected from healthy volunteers (ages 20–35 years) at the Warsaw Regional Blood Centre. Human neutrophils were isolated from buffy coats by dextran sedimentation and Pancol centrifugation [55], providing a neutrophil preparation with purity over 97%. Neutrophils were suspended in RPMI 1640 and stored at a temperature of 4 °C until subsequent analysis. 

#### 4.9.2. Evaluation of Neutrophils’ Viability

Flow cytometry using propidium iodide (PI) staining was undertaken to evaluate the influence of honokiol and magnolol on neutrophils’ viability, following a previously described method [56]. Briefly, neutrophils (2.0 × 10^6^/mL) were cultured for 24 h in RPMI 1640 medium (supplemented with 10% FBS, 1% penicillin–streptomycin, 10 mM HEPES and 2 mM L-glutamine) in the absence/presence of the tested compounds (12.5–50 µM), added 30 min before stimulation with LPS 100 ng/mL. After incubation, cells were centrifuged, washed and re-suspended in PI solution 0.5 μg/mL, followed by incubation in the dark at room temperature (15 min). Within 1 h, the neutrophils were analyzed by a BD FACSCalibur flow cytometer (BD Biosciences, San Jose, CA, USA). The cells showing high permeability to PI were considered PI+ cells. The neutrophils’ viability was calculated using the following formula: 100% − PI+ cells%. Triton X-100 (0.1%) was used as a positive control.

#### 4.9.3. Evaluation of IL-1β, IL-8 and TNF-α Secretion

The evaluation of cytokine release from LPS-stimulated neutrophils was assessed following a previously described protocol [56]. Thus, neutrophils were incubated at 37 °C in a 96-well plate in RPMI 1640 medium (prepared as described in Section 4.9.2.), with/without honokiol or magnolol (12.5, 25 and 50 µM) added 30 min prior to neutrophils’ stimulation with LPS 100 ng/mL. After 24 h, the plates were centrifuged, the supernatants were collected and the cytokines’ release was determined by ELISA assay kits following the manufacturer’s instructions (BD Biosciences, San Jose, CA, USA) using a Synergy 4 BioTek microplate reader (Winooski, VT, USA). The effect on cytokine production was determined through the percentage of released cytokines relative to the control without the test compounds. Urolithin A (a substance with known anti-inflammatory properties [57,58], previously acquired by isolation (>95% HPLC-DAD purity) in the Department of Pharmacognosy and Molecular Basis of Phytotherapy, Medical University of Warsaw, Warsaw, Poland) at concentrations of 12.5, 25 and 50 µM was used as a positive control.

### 4.10. Statistical Analyses

The results were calculated as mean ± standard error of the mean (SEM) of the indicated number of experiments. The one-way analysis of variance (ANOVA) with Tukey’s and Dunnet’s post-hoc tests was used to evaluate the statistical significance, which was set at *p*-values less than 0.05.

## 5. Conclusions

Our study shed light into the antidermatophytic effects of honokiol and magnolol. The antifungal screening revealed that both neolignans are highly active against dermatophytes and act as fungicidal agents. The mechanism of activity included the impairment of the ergosterol pathway, with accumulation of squalene in fungal cells. Binary mixtures of magnolol or honokiol with terbinafine showed synergistic and additive effects against *T. rubrum*, respectively. Moreover, both compounds influenced the cytokine generation by LPS in ex vivo human neutrophils: magnolol significantly inhibited IL-1β, IL-8 and TNF-α release, while honokiol decreased only IL-1β secretion. In conclusion, our results can be regarded as a starting point in the development of novel therapy strategies against *T. rubrum*-related dermatophytosis. 

## Figures and Tables

**Table 1:** The antifungal activity (MIC, MFC expressed as mg/L) of honokiol, magnolol and terbinafine against dermatophytes.

Strains	Honokiol		Magnolol		Terbinafine	
	MIC	MFC	MIC	MFC	MIC	MFC
*Trichophyton rubrum* ATCC 28188	8	16	8	16	0.031	0.031
*Trichophyton mentagrophytes* ATCC 9533	8	16	8	16	0.031	0.031
*Trichophyton rubrum* RTCC 1348 *	8	16	8	16	0.031	0.031
*Trichophyton rubrum* RTCC 2158 *	8	16	8	16	0.062	0.062
*Trichophyton ajelloi* RTCC 1356 *	8	16	8	16	0.062	0.062
*Microsporum gypseum* RTCC 2172 *	8	16	8	16	0.062	0.062
*Microsporum canis* RTCC 1883 *	8	16	8	16	0.062	0.062

ATCC = American Type Culture Collection; MIC = minimum inhibitory concentration; MFC = minimum fungicidal concentration. * Clinical isolates deposited into Romanian Type Culture Collection (RTCC) hosted by Iași University of Life Sciences (Romania).

**Table 2:** The minimum inhibitory concentration and fractional inhibitory concentration index of honokiol or magnolol in combination with terbinafine against *T. rubrum* ATCC 28188.

Combination	MIC * _in combination_ (mg/L)	FIC	FICI	Interpretation
Honokiol	0.5	0.06	0.56	Add
Terbinafine	0.015	0.50		
Magnolol	2	0.25	0.50	Syn
Terbinafine	0.007	0.25		

Add—addition; FIC—fractional inhibitory concentration; FICI—fractional inhibitory concentration index; Syn—synergism. * MIC of tested agent in the most effective combination.

## Data Availability

Not applicable.
